# NASA GeneLab derived microarray studies of *Mus musculus* and *Homo sapiens* organisms in altered gravitational conditions

**DOI:** 10.1038/s41526-024-00392-6

**Published:** 2024-04-26

**Authors:** Konstantinos I. Adamopoulos, Lauren M. Sanders, Sylvain V. Costes

**Affiliations:** 1 National Technical University of Athens, School of Electrical and Computer Engineering, Biomedical Engineering Laboratory, Zografou, Athens Greece; 2 Blue Marble Space Institute of Science, Space Biosciences Division, NASA Ames Research Center, Moffett Field, CA USA; 3 NASA Space Biosciences Division, NASA Ames Research Center, Moffett Field, CA USA

**Keywords:** Databases, Molecular biology

## Abstract

One of the greatest challenges of humanity for deep space exploration is to fully understand how altered gravitational conditions affect human physiology. It is evident that the spaceflight environment causes multiple alterations to musculoskeletal, cardiovascular, immune and central nervous systems, to name a few known effects. To better characterize these biological effects, we compare gene expression datasets from microarray studies found in NASA GeneLab, part of the NASA Open Science Data Repository. In this review, we summarize these archived results for various tissues, emphasizing key genes which are highly reproducible in different mice or human experiments. Such exhaustive mining shows the potential of NASA Open Science data to identify and validate mechanisms taking place when mammalian organisms are exposed to microgravity or other spaceflight conditions. Our comparative meta-analysis findings highlight certain degrees of overlap and reproducibility in genes identified as differentially expressed within musculoskeletal tissues in each species across a variety of altered gravity conditions. However, the level of overlap between species was found to be significantly limited, partly attributed to the limited availability of human samples.

## Introduction

NASA GeneLab platform, part of the NASA Open Science Data Repository (OSDR; https://osdr.nasa.gov/bio) constitutes an omics (e.g., genomics, transcriptomics, proteomics, metabolomics) interactive open-access database where users are able to upload, download, share, store and analyze data from spaceflight and space-relevant experiments. Approximately half of the datasets in the GeneLab repository consist of transcription profiling assays. The most prevalent transcription study assay type is microarray with RNA sequencing data being the fastest-growing assay in the database. Other assay types include genome sequencing, protein expression, metabolite profiling, epigenomics, metagenomics, and epitranscriptomics^1,2^.

There are still many unknown mechanisms regarding the biological effects taking place during spaceflight. Discovering these mechanisms is essential for NASA’s goal to keep its astronauts healthy, especially with the planned human deep space exploration missions, which are near the horizon. From the very first space missions, it became evident that microgravity causes physiological changes to mammals, including significant abnormalities presented in the cardiovascular, immune, vestibular and musculoskeletal systems^3,4^. There are multiple and intricate hazards to human health and performance in spaceflight caused by a combination of environmental stressors such as gravitational alterations, ionizing radiation, high level of carbon dioxide, changes in diet and physiological stress. Appropriate countermeasures must be developed in order to effectively minimize the aforementioned impairments^3,5–7^. Due to the small size of the astronaut cohort and privacy concerns surrounding human data collection and sharing, most spaceflight data originate from model organisms rather than *Homo sapiens*. An example of a model organism is *Mus musculus*, a suitable model for studying the effects of altered gravitational conditions. Current knowledge of the effects of spaceflight is derived from experimental models in vivo, ex vivo (e.g., tissue) and in vitro (e.g., cell culture)^8^.

Opportunities for actual microgravity experiments are rare and challenging to access due to the infrequency of spaceflights and the various associated restrictions, including expenses, equipment requirements, and the need for volunteers. Thus, space analogs, such as random positioning machines, clinostats, head-down bedrest, hindlimb unloading rodent model, and rotating wall vessel, have been developed aiming to simulate altered gravity conditions^9–11^.

In this study, we review the effects of microgravity and other spaceflight environmental factors on *Homo sapiens* and *Mus musculus* based on microarray studies and experiments housed in the NASA GeneLab repository^1,2^. We primarily focus on experiments that have been conducted in either actual or simulated microgravity in order to determine the affected functionalities at the gene, cellular or organ levels.

## Results

### Review of effects of altered gravitational conditions on Mus musculus

It has been shown that the central nervous system (CNS) of *Mus musculus* exhibits severe abnormalities after exposure to spaceflight. For example, neuroinflammation has been identified following ionizing radiation individually or combined with microgravity exposure^12,13^. Santucci et al. (OSD-33, 10.26030/7btg-6q49) studied the effects on gene and protein expression level in mouse brain tissue following a 3-month mission on the International Space Station (ISS). They found that the expression of nerve growth factor (Ngf) was decreased in hippocampus, cortex and adrenal gland during spaceflight conditions, whereas brain-derived neurotrophic factor (*Bdnf*) did not show consistent changes across the brain regions and the adrenal gland. Both proteins are involved in learning and memory via regulating neural plasticity. Genes related to various metabolic and catabolic processes were downregulated as well, while proteins related to mitochondrial and calcium metabolism, synthesis and hydrolysis of ATP, and amino acid transportation were upregulated^14^. Frigeri et al. (OSD-32, 10.26030/jpyz-fn46) performed mRNA expression analysis in brain tissue of mice that were hindlimb-unloaded for 2 weeks. Hindlimb unloading (HU) is a microgravity analog in which a rodent’s hind limbs are suspended and in disuse. Genes related to the transport of small molecules and ions into cells were upregulated in the HU group. An increased risk of venous thrombosis, as well as changes in functional pathways such as immune response, learning and memory performance, and cell junction were also noted^15^. Holley et al. (OSD-536, 10.26030/bg5q-t229) highlighted significant alterations in gene expression profiles associated with neuronal function, immune regulation, growth and metabolic function in brain tissue of mice that were housed on the ISS for thirty-five days. In particular, genes supporting neuronal synaptic signaling and migration were significantly downregulated^16^.

The detrimental consequences of microgravity exposure on the musculoskeletal system have also been extensively documented. Fitzgerald et al. (OSD-232, 10.26030/9626-w275) studied articular and sternal cartilage from mice that had experienced spaceflight conditions for 30 days. Genes that encode structural extracellular matrix components, such as *Fmod*, *Ogn*, *Omd*, *Dcn*, *Dpt*, *Prelp*, *Col10a1*, *Tsp4*, and *Comp* were downregulated in articular cartilage. Proteoglycan levels were lowered in the spaceflight articular cartilage, while there was no proteoglycan loss in the sternal cartilage. The authors concluded that this is because in microgravity, articular cartilage experiences near-complete biomechanical unloading, but sternal cartilage still experiences biomechanical loading^17^. Gambara et al. (OSD-135, 10.26030/rjyq-x751) found that in longissimus dorsi from mice flown for 30 days on the BION-M1 biosatellite, genes linked to insulin sensitivity and metabolism of skeletal muscle were significantly dysregulated, while myofiber cross-sectional area and myosin heavy chain subtype patterns were not altered^18^. Chakraborty et al. (OSD-396, 10.26030/ce4f-xx71) examined the procedure of bone fracture healing (using a segmental bone defect model) in mice housed on the ISS for 4 weeks. Micro-computed tomography (μCT) analysis of callus tissue after spaceflight indicated increased trabecular spacing and decreased trabecular connectivity, while an apoptosis and cellular morbidity gene-network signal was activated in the spaceflight callus tissue compared to controls^19^. Gambara et al. (OSD-111, 10.26030/9580-9n52) assessed changes in gene expression in mouse soleus and extensor digitorum longus following 30 days on the BION-M1 capsule. They identified differentially expressed genes related to key biological processes such as contractile machinery, calcium homeostasis, muscle development, cell metabolism, and inflammatory and oxidative stress response^8^. Based on data from the same mice, Blottner et al. studied the *Homer* gene, which is thought to be downregulated during muscle atrophy. They found the short *Homer1a* isoform was upregulated, while the long *Homer2* isoform was downregulated in the soleus muscle after microgravity exposure. This isoform sensitivity appears to be regulated by muscle activity or inactivity at the neuromuscular junction^20^. Däpp et al. (OSD-228, 10.26030/bfmx-z866) examined transcriptome alterations in soleus tissue of mice that underwent hindlimb suspension (HLS). HLS is a microgravity analog in which a rodent’s hind limbs are suspended, resulting in disuse similar to microgravity exposure. They found increased myogenic factors, contractile genes, and metabolic genes during reloading, as well as indications that sarcomere regulation is sensitive to mechanic loading^21^. Flück et al. recommended that especially in tissue plasticity studies, we should normalize gene expression levels using tissue-relevant references (e.g., muscle weight, volume, nuclear content)^21,22^. Mazzati et al. (OSD-227, 10.26030/vk0m-0558) also used HLS to detect differential expression of genes associated with lipid and glucose metabolism in soleus and gastrocnemius tissue^23^. Allen et al. (OSD-21, 10.25966/c36b-3g68) stated that spaceflight significantly alters expression of gastrocnemius tissue genes that are associated with muscle growth (e.g., *phosphatidylinositol 3-kinase regulatory subunit p85a*, *MAFbx/atrogin1*, *insulin response substrate-1*, and *forkhead box O1 transcription factor*) and muscle fiber type^24^.

Furthermore, weightlessness causes a decrease in bone formation by osteoblasts and an increase in osteolytic functions of osteoclasts. Pardo et al. examined gene expression profiles of 2T3 preosteoblast cells to find genes particularly sensitive to gravity changes, and found that simulated microgravity downregulated alkaline phosphatase, run-related transcription factor 2, osteomodulin and parathyroid hormone receptor 1, while cathepsin K was upregulated^25^. Patel et al. (OSD-30, 10.25966/90vx-bf79) compared preosteoblast exposure to two different simulators of altered gravity: Rotating Wall Vessel and Random Positioning Machine. They found similar results from each method, including altered expression of the same 14 skeletal remodeling genes. In particular, they found inhibited alkaline phosphatase enzyme activity in both, demonstrating that both methods inhibit preosteoblast differentiation, consistent with microgravity-induced bone mass decrease. Parathyroid hormone-related protein (PthR1) involved in calcium mobilization and bone morphogenetic protein 4 (BMP4) associated with skeleton development (e.g., cartilage formation) were downregulated in the Rotating Wall Vessel conditions^26^. The role of parathyroid hormone-related protein was also studied by Camirand et al. (OSD-107, 10.26030/2x2z-6w28) using trabecular and calvarial cells. They confirmed that PthR protein has anti-apoptotic function under microgravity, and as a result it could be defined as an anabolic agent to prevent cell death in trabecular osteoblasts^27^. Uda et al. (OSD-324, 10.26030/s7kj-h383) studied altered gravity effects on osteocytic cell line Ocy454 for 2, 4 and 6 days onboard the ISS, concluding that glucose metabolism and oxygen consumption were increased during spaceflight^28^. Wang et al. (OSD-547, 10.26030/y2af-c498) examined gene expression profiles of osteocyte-like cell line MLO-Y4 under simulated altered gravitational conditions via large gradient high magnetic field, showing that this environment affected the expression of genes related to enzyme, peptide, G-protein coupled receptors and glucose metabolic process^29^. Sambandam et al. (OSD-18, 10.25966/j6hy-d340) investigated the differentiation procedure of osteoclasts using a rotary cell culture system to simulate microgravity. In particular, they highlighted increased expression of genes associated with enhanced osteoclast differentiation and function, such as cytokines/growth factors, proteases and signaling proteins^30^.

Osteoblast precursors such as Bone Marrow Stromal Cells (BMSC) are sensitive to mechanical loading. Monticone et al. (OSD-29, 10.25966/6rr8-r017) studied murine BMSC cultures that were onboard the ISS for 8 days, while half of them were stimulated with osteo-inductive medium. They found that cell proliferation was inhibited in spaceflight conditions and that differentially expressed genes were related to neural development, neuron morphogenesis, transmission of nerve impulse and synapse^31^. Ortega et al. (OSD-50, 10.26030/69kd-nx87) differentiated murine bone marrow cells (mBMC) in the presence of recombinant macrophage colony-stimulating factor (rM-CSF) for 14 days during spaceflight, detecting significant increase in cell proliferation and differential expression of genes related to the coagulation pathway. They found that the macrophages that differentiate during spaceflight express slightly different markers, and that the post-spaceflight population may be slightly more differentiated^32^. In addition, Chapes and Ortega (OSD-50, 10.26030/69kd-nx87) highlighted the importance of ground-based experiments before spaceflight in order to assess macrophage differentiation^33^.

Astronauts suffer from skin dryness, itching, coarsening of epidermis and decreased skin elasticity. Neutelings et al. (OSD-61, 10.26030/2zjp-sj35) reported a fifteen percent reduction of dermal thickness of cutaneous tissue collected from mice exposed to spaceflight conditions for 90 days. They also reported deregulated hair follicle cycle and upregulated myogenesis^34,35^. Mao et al. (OSD-116, 10.26030/ya5a-e896) confirmed the increased risk for pathophysiology damage and carcinogenesis in skin tissue during a 13-day spaceflight mission as they observed upregulation in cellular antioxidants, ROS production and tissue remodeling^36^.

Weightlessness induces severe effects on the immune system as well. Lebsack et al. (OSD-4, 10.25966/qq9p-pc28) reported alterations in the expression of genes regulating stress, T-cell signaling activity and glucocorticoid receptors following the exposure of mice to spaceflight for 13 days. In particular, *Rbm3*, *Ctla-4*, *IFN-a2a* genes were upregulated and *Hsph110*, *Hsp90aa1*, *Cxcl10*, *Strip1*, *Fkbp4m*, and *CD44* genes were downregulated in the thymus tissue of spaceflown mice^37^.

Beheshti et al.^38^ utilized the NASA GeneLab database to analyze transcriptomic data from different rodent datasets (e.g., OSD-4, 10.25966/qq9p-pc28, OSD-21, 10.25966/c36b-3g68, OSD-25, 10.25966/kzxa-s692, OSD-48, 10.26030/jq04-0n51, OSD-61, 10.26030/2zjp-sj35, OSD-63, 10.26030/gsmt-8e70, OSD-111, 10.26030/9580-9n52), revealing critical genes, signaling pathways and circulating micro-RNA signatures as key biomarkers for astronauts’ health. Particularly, they identified *TP53* and transforming growth factor beta one (*TGF-β1*) as the most prevalent regulators across all tissues, and *TGF-β1* was the most connected gene across all tissues, suggesting it is a key driver gene for spaceflight response^34^.

### Review of effects of altered gravitational conditions on Homo sapiens

Experiments of human muscle tissue exposed to microgravity validated the aforementioned findings concerning the musculoskeletal system’s vulnerability to altered gravity. Chopard et al. (OSD-51, 10.26030/3w73-jn41) examined gene expression profiles of soleus and vastus lateralis muscles during long-term (60 days) bedrest conditions, as well as evaluating potential countermeasures such as protein supplementation and combined resistance-aerobic exercise. They reported an induction of metallothioneins, genes involved in antioxidant stress response, in the non-countermeasures groups. They also reported limited counteracting effects of the nutritional countermeasure, while aerobic training significantly counteracted the negative effects on muscle metabolism^39^. Rullman et al. (OSD-198, 10.26030/kcrr-p336) added the hypoxia variable into the equation, studying effects on micro-RNA (miRNA) expression of horizontal bedrest with normal level of oxygen, bedrest in hypoxia, and ambulation in hypoxia. Few miRNAs were modestly differentially regulated, such as *let-7*, *miR-15*, *miR-25*, *miR-199*, and *miR-133*, thus only minor alterations could be detected. The Planetary Habitat Simulation study aimed to clarify biological effects of prolonged (21-day) musculoskeletal unloading combined with hypoxia. Rullman et al. (OSD-195, 10.26030/r6bv-rk07) identified upregulation of genes associated with denervation (e.g., acetylcholine receptor subunit delta and perinatal myosin) and a robust inhibition of the myocyte enhancer factor-2 (*MEF2*) family^40,41^. Alibegovic et al. (OSD-370, 10.26030/zyy0-6497) investigated muscle biopsies taken from healthy young men before and after bedrest, in basal or insulin-stimulated states collected from vastus lateralis as well. They confirmed downregulation of mitochondrial function pathways and increased muscle insulin resistance induced by bedrest conditions, which were only partially recovered after muscle retraining^42^.

In addition, several studies have aimed to determine how human bone marrow stem cells react and differentiate in altered gravitational conditions. Mayer-Wagner et al. (OSD-124, 10.26030/6fwd-2p79) studied how chondrogenesis is affected during simulated reduced gravity and low-frequency electromagnetic fields, concluding that microgravity conditions significantly downregulated *COLXA1*, *COL2A1*, and aggrecan, representing decreased chondrogenic potential^43^. Bradamante et al. (OSD-546, 10.26030/mg6a-qv31) examined the effects in terms of growth and differentiation of human bone marrow stem cells (hBMSCs) housed on the ISS for 2 weeks. They identified upregulation of genes that are related to osteogenesis (*BGLAP*, *CHRDL1*, and *SPP1*) and metabolism of steroid hormones and vitamins A, D pathway (*CYP19A1*, *CYP24A1*, *AKR1B1*, *HSD11B1*). Cell proliferation, motility and cell-cell communication seemed also to be affected as evidenced by downregulation of *CSNK2A2*, *ITGAV*, *NRCAM*, and *NRP2*, while the *RAB27b* gene associated with microvesicle formation was significantly upregulated. Moreover, they reported stemness loss, no evidence of apoptosis or senescene, notable upregulation of collagen genes, and notable downregulation of matrix metallopeptidases^44^.

Terada et al. (OSD-174, 10.26030/6sg0-ng36) analyzed data from a JAXA (Japan Aerospace Exploration Agency) experiment conducted on a 6-month mission on the ISS, where both hair follicles and shafts were collected from ten astronauts. They reported altered expression of genes associated with cell cycle disruption in hair follicles (e.g., *COMP* and *CDK1*) and hair growth (e.g., *ANGPTL7* and *FGF18*)^45^. Zhang et al. (OSD-118, 10.26030/rcyt-qp10) investigated miRNA expression in non-proliferating human fibroblast cells in response to spaceflight environment (ISS). Minor effects were observed on gene or miRNA expression on day 14, whilst most of the differentially expressed genes were related to cell growth on day 3 (see ref. ^46^). Lu et al. (OSD-114, 10.26030/yssj-bg68) studied human fibroblasts housed on ISS with and without treatment of bleomycin (a compound which induces DNA damage), to better understand the effects of microgravity on cellular DNA damage response. Although several genes were altered between treatment and control groups in flight and on Earth, including some of the same genes, there was no significant change when comparing flight treatment to Earth treatment. The authors also concluded that cell type and cell growth condition influence whether microgravity affects DNA damage response^47^.

Spaceflight causes profound effects on lymphocyte functions that are well documented. Ward et al. (OSD-5, 10.25966/qq4z-4m04) detected ten downregulated genes (e.g., *GNLY*, *PSME2*, *PrX4*, *HLA-DRA*, *LY75*, *IL18*, and *DOCK2*) related to immune response in activated T lymphocytes that were exposed to simulated microgravity (Rotating Wall Vessel) for 24 h^48^. Chang et al. (OSD-13, 10.26030/4an8-r968) studied T cells housed on the ISS and stimulated with the T-cell mitogen ConA and anti-CD28. They found impaired T-cell activation as well as profound downregulation of *Rel/NF-κB*, *CREB*, *SRF* and immediate early genes’ expression, indicating very early effects of microgravity on T-cell gene expression^49^. Boonyaratanakornkit et al. (OSD-484, 10.26030/semj-9y19) revealed that Protein Kinase A (PKA) is a key regulator factor regarding *NF-κB*, *AP-1*, *CREB* and T-cell activation in simulated microgravity^50^. Thiel et al. (OSD-189, 10.26030/r0md-de60, OSD-172, 10.26030/jx41-b816, OSD-188, 10.26030/3jq1-s218) identified *ATP6V1A/D*, *IGHD3-3/IGHDE-10*, and *LINC00837* as genes that are significantly affected by gravity, as they investigated non-activated human Jurkat T cells in both microgravity and hypergravity conditions. They also found that chromosomal region 11p15.4 seems to be particularly robust to altered gravity^51,52^. Vogel et al. and Tauber et al. focused on the effects of altered gravity on immune signaling, as captured by the OSD-283 (10.26030/ge2v-wr94) and OSD-297 (10.26030/bb5k-1h18) datasets. In particular, Vogel et al. identified hypoxia-inducible factor 1 (HIF1) as a potential pharmacological target for counteracting deterioration of immune system, and *PDK1* as sensitive in Jurkat T and U937 myelomonocytic cells exposed to altered gravitational conditions. In addition, Tauber et al. described well-regulated homeostasis and transcriptional stability of oxidative stress-related pathways^53,54^. U937 myelomonocytic cells were also studied by Thiel et al. (OSD-283, 10.26030/ge2v-wr94, OSD-297, 10.26030/bb5k-1h18), who detected significant gene expression alterations after only 20 s of altered gravitational conditions (both microgravity and hypergravity). However, all initially differentially expressed transcripts adjusted rapidly afterward, verifying the existence of immediate adaptation mechanisms^55^. Long noncoding RNAs (lncRNAs) and miRNAs seem to regulate effects, associated with apoptosis and immune response, in human lymphoblastoid TK6 cells under simulated microgravity and ionizing radiation according to Fu et al. (OSD-545, 10.26030/7ta4-ga35)^56^.

Chakraborty et al. (OSD-54, 10.26030/8mfb-wa73) customized a cell culture module for human dermal microvascular endothelial cell growth in order to examine the impact of spaceflight environment on endothelial cells treated with lipopolysaccharide. They found that long-term lipopolysaccharide exposure resulted in a delayed host response, inefficient in pathogens’ potential invasion^57^. Human umbilical vein endothelial cells’ exposure to microgravity showed significant altered transcripts related to oxidative phosphorylation, stress response, cell cycle and apoptosis. Findings from Versari et al. (OSD-52, 10.26030/nt3p-p547) suggested that microgravity affects inflammatory response and endothelial behavior, while promoting senescence^58^.

Girardi et al. (OSD-55, 10.26030/9thk-dv75, OSD-56, 10.26030/nc96-xp67, OSD-128, 10.26030/73sd-9a85, OSD-129, 10.26030/sec3-y188) examined mRNA and miRNA expression profiles in peripheral blood lymphocytes (PBL) exposed to simulated microgravity through a miRNA-mRNA integration analysis, detecting effects on inflammatory response, apoptosis and cell proliferation decrease^59^. *Let-7i, miR-7, miR-7-1, miR-27a, miR-144, miR-200a, miR-598*, and *miR-650* were found to be deregulated in human PBLs exposed to radiation and simulated microgravity (Rotating Wall Vessel)^60^. Wei et al. also investigated effects of simulated weightlessness on human PBLs. Microgravity seemed to inhibit DNA replication and down-regulate DNA-repair gene, enhancing structural chromosome instability^61^. Moreover, Fuentes et al. found that the effect of microgravity in cardiovascular progenitors is age-dependent, since endothelial and cardiomyogenic differentiation markers were highly expressed in adults whereas neonatal progenitors acquired dedifferentiating cellular features^62^. In a recent study, Bisserier et al. investigated spaceflight effect on small extracellular vesicles (sEVs) isolated from three astronauts’ blood plasma. Significant downregulation of *miR-214*, *miR-128* and promotion of PRC2 complex activation and elevated *H3K27me3* levels were observed in human cardiomyocytes during spaceflight, leading to epigenetic suppression of Vitamin D receptor expression^63^.

The effects of altered gravitational conditions on lymphoblast leukemic cells and colorectal cancer cells were studied by Vidyasekar et al. (OSD-125, 10.26030/6dnk-x507). They reported multiple microgravity-induced consequences observed in both cell lines such as reduced cell viability, altered cell morphology and diverging cell cycle. Dysregulation of oncogenes and cancer progression markers (*JUNB*, *CD44*, *MYC* and *CD117*) were detected as well^64^.

### Comparative analysis of microgravity effects on musculoskeletal systems

We present a differential gene expression analysis in response to microgravity vs control conditions. To gain insight into the molecular mechanisms underlying these conditions we generated volcano plots per each tissue which depict both the statistical significance and the level of differentially expressed genes (DEGs). In addition to that, we evaluated the Gene Ontology functions of the top over-expressed and under-expressed genes.

**Fig. 1:**
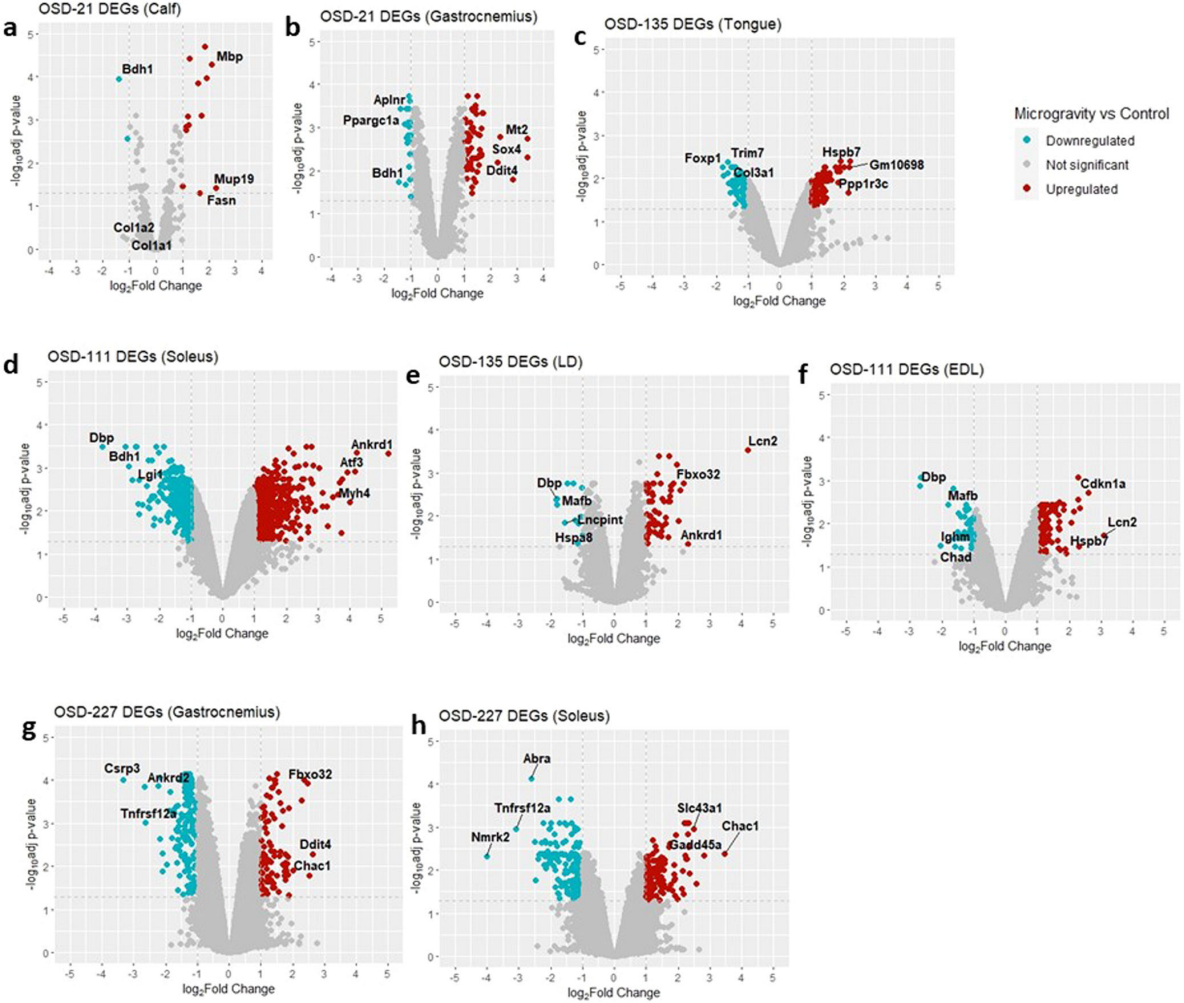
Volcano plots showing gene expression between microgravity and control conditions in mouse muscle datasets. **a** OSD-21 calf; **b** OSD-21 gastrocnemius; **c** OSD-125 tongue; **d** OSD-111 soleus; **e** OSD-135 longissimus dorsi; **f** OSD-111 extensor digitorum longus; **g** OSD-227 gastrocnemius; **h** OSD-227 soleus. Genes with a log2-fold change above 1 are represented in red, signifying upregulated expression, while genes with a log2-fold change below −1 are depicted in blue, indicating downregulated expression. The horizontal dashed line corresponds to the adjusted *P* value threshold of less than 0.05, highlighting genes with statistically significant expression changes.

Figure 1 shows volcano plots of all musculoskeletal mouse muscle microarray datasets from OSDR. These plots show the variation and diversity of differential gene expression results even between the same types of muscle. For example, Fig. 1b, g are both from gastrocnemius datasets but the differentially expressed genes and distribution are very different.

**Fig. 2:**
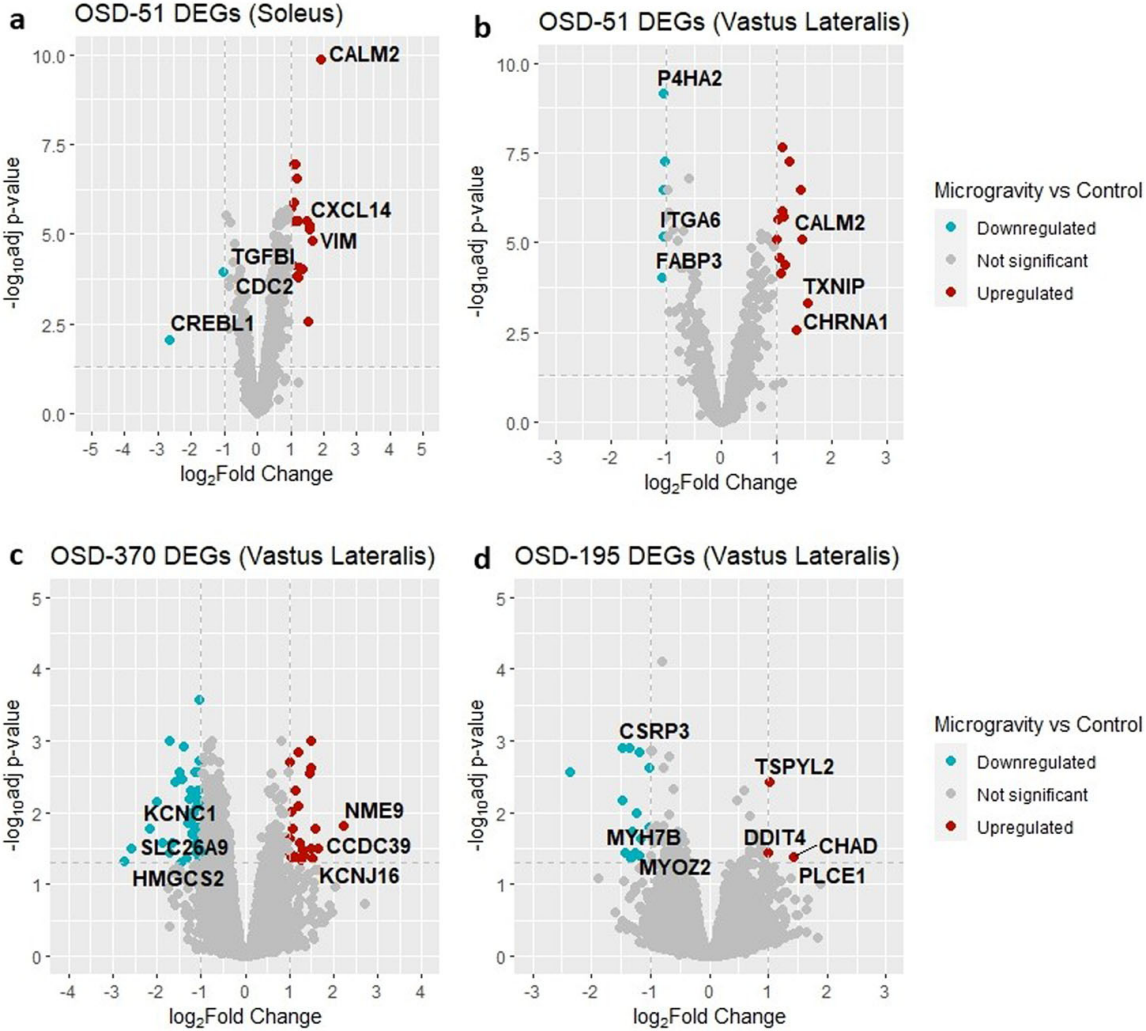
Volcano plots showing gene expression between microgravity and control conditions in human muscle datasets. **a** OSD-51 soleus; **b** OSD-51 vastus lateralis; **c** OSD-370 vastus lateralis; **d** OSD-195 vastus lateralis. Genes with a log2-fold change above 1 are represented in red, signifying upregulated expression, while genes with a log2-fold change below −1 are depicted in blue, indicating downregulated expression. The horizontal dashed line corresponds to the adjusted *P* value threshold of less than 0.05, highlighting genes with statistically significant expression changes.

Figure 2 shows volcano plots for all human musculoskeletal microarray datasets from OSDR. Similar to the mouse datasets, there is widespread variation in both distribution and differentially expressed genes between datasets of the same tissue type.

**Fig. 3:**
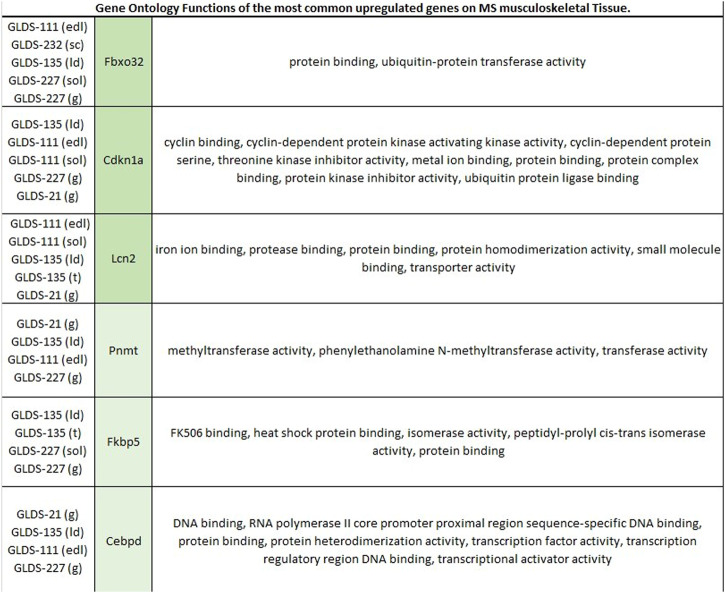
Gene Ontology functions of all differentially expressed genes upregulated in four or more tissue types in mouse musculoskeletal datasets. Dark green shadowed genes are differentially expressed in five tissue types; light green shadowed genes are differentially expressed in four tissue types. sc sternal cartilage, ld longissimus dorsi, sol soleus, edl extensor digitorum longus, g gastrocnemius.

**Fig. 4:**
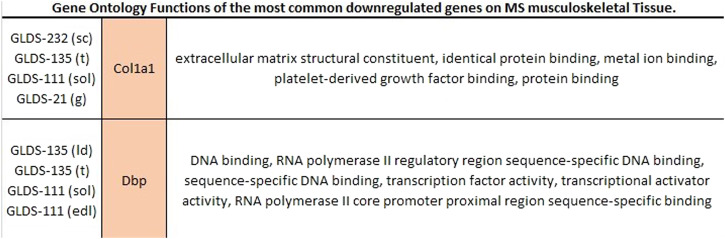
Gene Ontology functions of all differentially expressed genes downregulated in four tissue types in mouse musculoskeletal datasets. sc sternal cartilage, ld longissimus dorsi, sol soleus, edl extensor digitorum longus, g gastrocnemiussc sternal cartilage, t tongue, ld longissimus dorsi, sol soleus, sol soleus, g gastrocnemius, edl extensor digitorum longus.

**Fig. 5:**
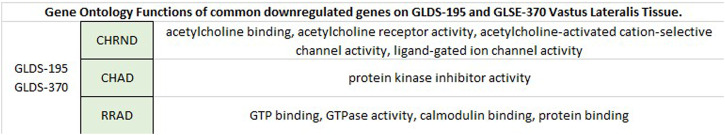
Gene Ontology functions of differentially expressed genes upregulated in two human musculoskeletal datasets. *CHRND, CHAD*, and *RRAD* upregulated genes in *Homo sapiens*’ vastus lateralis tissues appear to be involved in multiple enriched GO terms.

**Fig. 6:**
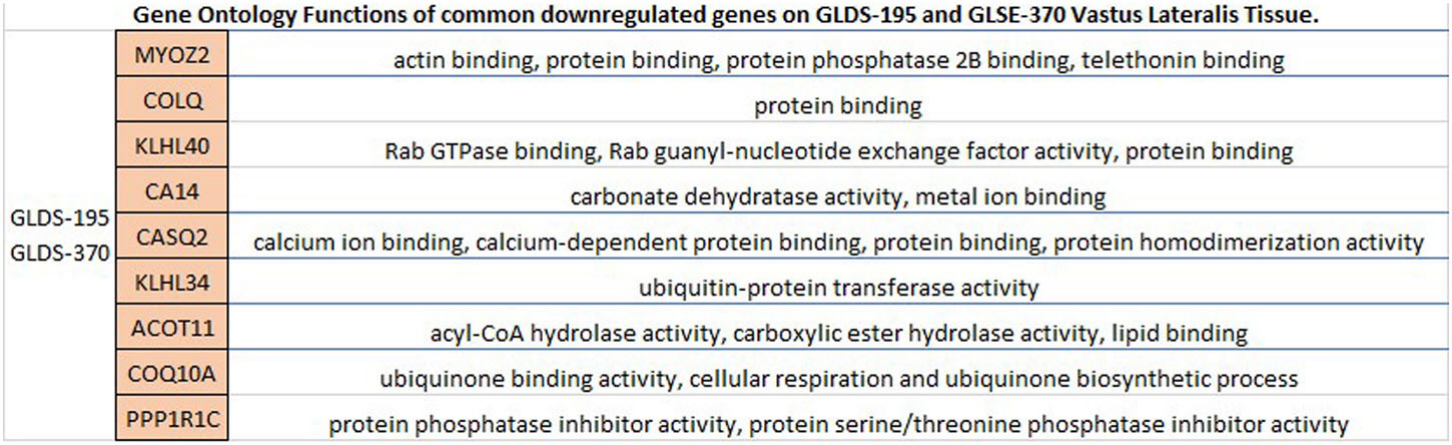
Gene Ontology functions of all differentially expressed genes downregulated in two human musculoskeletal datasets. *MYOZ2, COLQ, KLHL40, CA14, CASQ2, KLHL34, ACOT11, COQ10A,* and *PPP1R1C* downregulated genes in *Homo sapiens*' vastus lateralis tissues appear to be involved in multiple enriched GO terms.

We then evaluated the Gene Ontology functions of the genes that were significantly upregulated in four or more mouse musculoskeletal tissues across experiments: *Fbxo32, Cdkn1a, Lcn2, Pnmt, Fkbp5,* and *Cebpd* (Fig. 3). We also evaluated the Gene Ontology functions of the genes that were significantly downregulated in four mouse musculoskeletal tissues across experiments: *Col1a1* and *Dbp* (Fig. 4). We performed a similar analysis using all datasets from human musculoskeletal tissue (Figs. 5 and 6).

We further expanded the analysis by incorporating all available data from literature derived from the same tissue and organism, utilizing similar experimental conditions, without imposing any specific inclusion criteria (e.g. *P* value, log2-fold change). Consequently, we proceeded with the computation of Pearson correlation coefficients for all these genes across experiments that evaluated comparable factors. First, we investigated the concordance of gene expression levels between two male human bedrest experiments studying vastus lateralis tissue (OSD-195 and OSD-370). The most notable difference between these two datasets is that OSD-195 studied 21 days of bedrest while OSD-370 studied 9 days of bedrest. The correlation coefficient was calculated to assess the gene-by-gene relationship between the two datasets. Our analysis revealed a moderate positive correlation with a coefficient of 0.47. This indicates that there is a tendency for the gene expression levels to increase together across the two experiments.

**Fig. 7:**
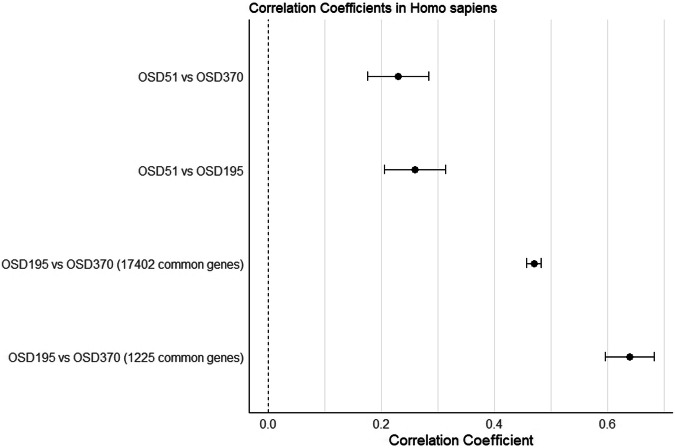
Forest plot depicting the correlation coefficients for four comparisons including OSD-195 vs OSD-370, OSD-51 vs OSD-195, OSD-51 vs OSD-370, and OSD-195 vs OSD-370 from a larger merged dataset (17402 genes) in Homo sapiens. The correlation coefficient for each comparison is represented by a black point on the line. The dashed vertical line at the center denotes the point of no correlation (correlation coefficient = 0). The confidence level employed for computing the confidence intervals (CIs) is configured to be 95%, equivalent to an alpha level of 0.05.

We extended our investigation to include the OSD-51 experiment, female human bedrest study which shared 1225 common unique genes with both OSD-195 and OSD-370 experiments from the larger merged list of 17,402 genes. For OSD-51 compared to OSD-195, a correlation coefficient of 0.26 was observed, indicating a weak positive correlation between the gene expression levels of the two experiments. Similarly, between OSD-51 and OSD-370, a correlation coefficient of 0.23 was found, also indicating a weak positive correlation. However, notably, the correlation between OSD-195 and OSD-370 experiments was significantly higher, with a coefficient of 0.64, even using the reduced number of genes, indicating a moderate positive correlation. This suggests that these two experiments exhibit more similar gene expression patterns when compared to OSD-51. The increase in correlation between OSD-195 and OSD-370 compared to the larger merged dataset (Fig. 7).

We then examined the correlation coefficient between two mouse gastrocnemius datasets (OSD-21 and OSD-227), focusing on 12,385 unique common genes. The greatest difference between these two experiments is that OSD-21 studied female mice flown on an 11-day space shuttle flight while OSD-227 studied male mice subjected to hindlimb suspension for 24 h. The calculated correlation coefficient was close to 0.2, falling within the range of a weak positive correlation.

**Fig. 8:**
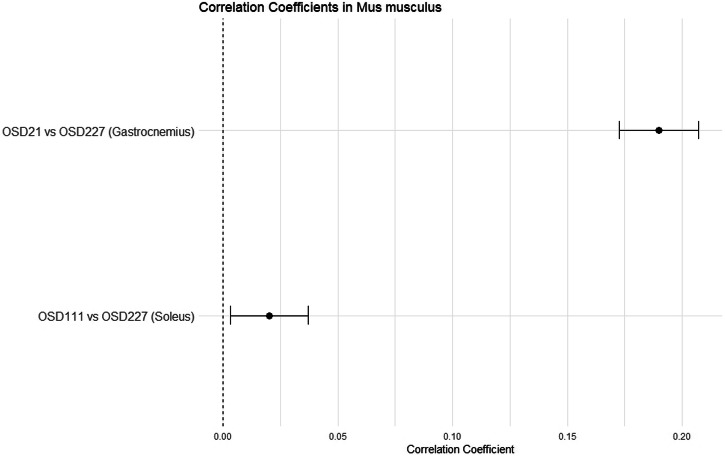
The figure displays the correlation coefficients for two comparisons including OSD-21 vs OSD-227 (Gastrocnemius), OSD-111 vs OSD-227 (Soleus) in Mus musculus. The correlation coefficient for each comparison is represented by a black point on the line. The dashed vertical line at the center denotes the point of no correlation (correlation coefficient = 0). The confidence level employed for computing the confidence intervals (CIs) is configured to be 95%, equivalent to an alpha level of 0.05.

We also calculated the correlation coefficient between OSD-227 soleus and OSD-111 soleus, where OSD-111 studied male mice flown on the BION-M1 biosatellite for 30 days. The Pearson correlation coefficient calculated for the shared genes between OSD-111 and OSD-227 was found to be 0.02 (Fig. 8). This correlation coefficient indicates minimal linear relationship between the gene expression patterns in the two experiments.

Overall, we observe higher correlation coefficient between datasets which studied subjects of the same sex under similar experimental conditions. Several factors can contribute to the correlation coefficient, including biological variability, technical factors, and the complexity of gene expression regulation. Even with the same tissue and similar experimental conditions, gene expression can exhibit considerable variation between samples and experiments.

## Discussion

Studies from spaceflight and ground analog experiments confirmed that altered gravity environment causes multiple physiological alterations to both mouse and human musculoskeletal, immune, cardiovascular, vestibular, integumentary, and nervous system, including biological processes such as metabolism of skeletal muscle, bone formation response, inflammatory response, apoptosis and cell proliferation in PBLs, impaired activation of T cells, stem cell differentiation, cell cycle disruption in hair follicles, neuron morphogenesis, neuronal synaptic signaling and migration, cell morphology and diverging cell cycle in cancer cell lines.

In our study, we initially incorporate all available literature data derived from the same tissue and organism with similar experimental conditions, without considering specific inclusion criteria. Subsequently, we computed Pearson correlation coefficients for all genes across experiments that evaluated comparable factors. Initially, we focused on *Homo sapiens’* Vastus lateralis tissue. The analysis revealed a moderate positive correlation indicating a tendency for gene expression levels to increase together across OSD-195 and OSD-370 experiments. We expanded our investigation to include the OSD-51 experiment. We observed a correlation coefficient of 0.26, showing a weak positive correlation between the gene expression levels between OSD-51 vs OSD-370, and OSD-51 vs OSD-195. However, the correlation between OSD-195 and OSD-370 experiments was notably higher, with a coefficient of 0.64, suggesting a more pronounced association and similarity in gene expression patterns when compared to OSD-51. The increased correlation between OSD-195 and OSD-370, despite a reduced number of common genes, indicates potential biologically relevant similarities between these two experiments, which may not be evident when considering the larger gene set. The calculated correlation coefficient between experiments regarding *Mus musculus* Gastrocnemius and Soleus tissues indicated weak positive and close to zero correlation, respectively.

Furthermore, we were interested in characterizing how reproducible these experimental results are within each species. We focused on the musculoskeletal system for a comparative analysis because this physiological system contains the greatest number of comparable tissue types between human and mouse datasets. We evaluated the Gene Ontology functions of the genes that were significantly upregulated in 4 or more mouse musculoskeletal tissues across experiments: *Fbxo32, Cdkn1a, Lcn2, Pnmt, Fkbp5,* and *Cebpd*. This analysis revealed a consistent trend of upregulation of several biological processes including protein binding, ubiquitin activity and transferase activity in mouse muscle tissues exposed to microgravity. We also evaluated the Gene Ontology functions of the genes that were significantly downregulated in two mouse musculoskeletal tissues across experiments: *Col1a1* and *Dbp*. This analysis shows consistent downregulation of several biological processes across experiments, including metal ion binding, DNA binding and RNA polymerase activity. Interestingly, several of the same biological processes were found to be associated with both upregulated genes and downregulated genes.

We performed a similar analysis using all datasets from human musculoskeletal tissue (Figs. 5 and 6). There were fewer datasets to compare, and three upregulated and nine downregulated genes overlapped between datasets. The *CHRND* gene (associated with acetylcholine binding, acetylcholine receptor activity, acetylcholine-activated cation-selective channel activity, ligand-gated ion channel activity), *CHAD* gene (related to protein kinase inhibitor activity), and *RRAD* gene (associated with GTP binding, GTPase activity, calmodulin binding, protein binding) were significant upregulated on two different human experiments, OSD-195 and OSD-370 both studying vastus lateralis tissue. The aforementioned experiments also share nine common downregulated genes *MYOZ2* (associated with actin, and protein binding), *COLQ* (associated with protein binding), along with *KLHL40, CA14, CASQ2, KLHL34, ACOT11, COQ10A,* and *PPP1R1C*. Overall, in the human musculoskeletal datasets we find consistency in the biological process of protein binding. OSD-195 and OSD-370 have the greatest overlap, even though the other two datasets also studied the same tissue type.

It was challenging to perform a direct comparison between the human and mouse results, due to the small sample size in the human datasets. However, we did note that the protein binding Gene Ontology term was related to several upregulated mouse and downregulated human genes.

We assess gene expression results from a variety of tissues in both *Mus musculus* and *Homo sapiens* exposed to altered gravity in spaceflight or ground analogs. We report some overlap and reproducibility in genes identified as differentially expressed in musculoskeletal tissues within each species, and very limited overlap between species, partially due to the small amount of human samples. To ensure the comprehensiveness of our analysis, we took an inclusive approach by incorporating all relevant literature data derived from the same tissue and organism, with similar experimental conditions. This inclusive approach may help to identify potential patterns or trends across a wider range of studies, which may not be apparent when focusing solely on a subset of data. The elevated correlation observed between OSD-195 and OSD-370, despite a reduced number of shared genes, implies the presence of potential biologically relevant similarities between these two experiments. Further research is needed to understand which differentially expressed gene signatures from *Mus musculus* are truly reflective of *Homo sapiens* response to altered gravity.

## Methods

### Data and literature mining

We identified all the microarray studies and datasets housed in NASA GeneLab, part of the NASA Open Science Data Repository (https://osdr.nasa.gov/bio) by applying the following search filters: “Assay Type: Microarray”, “Tissue: All”, “Factor: All”, “Organisms: *Mus musculus* OR Human (Homo sapiens)”. The search was limited to studies published up to January 2023 to ensure the inclusion of the most recent research.

We obtained the following datasets: OSD-4, OSD-18, OSD-21, OSD-29, OSD-30, OSD-32, OSD-33, OSD-50, OSD-61, OSD-79, OSD-80, OSD-87, OSD-88, OSD-89, OSD-93, OSD-94, OSD-107, OSD-109, OSD-111, OSD-116, OSD-117, OSD-131, OSD-135, OSD-148, OSD-153, OSD-156, OSD-158, OSD-159, OSD, 160, OSD-183, OSD-202, OSD-222, OSD-232, OSD-227, OSD-228, OSD-324, OSD-342, OSD-396, OSD-432, OSD-455, from *Mus musculus* and OSD-5, OSD-9, OSD-13, OSD-51, OSD-52, OSD-54, OSD-55, OSD-56, OSD-71, OSD-73, OSD-78, OSD-92, OSD-114, OSD-115, OSD-118, OSD-124, OSD-125, OSD-128, OSD-129, OSD-130, OSD-140, OSD-149, OSD-152, OSD-151, OSD-154, OSD-155, OSD-157, OSD-172, OSD-174, OSD-175, OSD-176, OSD-178, OSD-182, OSD-188, OSD-189, OSD-195, OSD-198, OSD-283, OSD-285, OSD-297, OSD-317, OSD-354, OSD-367, OSD-368, OSD-369, OSD-370, OSD-410, OSD-484, OSD-542, OSD-544, OSD-545, OSD-546 from *Homo sapiens*.

Furthermore, the GeneLab repository federates search with the Gene Expression Omnibus (GEO) of National Center for Biotechnology Information (NCBI). The GeneLab repository encompasses the entirety of the relevant datasets present in the GEO database. A more comprehensive search had to be done though using the following query: ”(microgravity OR spaceflight OR gravity) AND “Homo sapiens”) and ”(microgravity OR spaceflight OR gravity) AND “Mus musculus”) and applying the following filter criteria: “Study type: Expression profiling by array”. Academic research databases, such as PubMed, and ScienceDirect were also used with the keywords “microgravity and microarray”, “spaceflight and microarray”, “gravity and microarray” and “weightlessness and microarray”. Because GeneLab ingests datasets from GEO and provides an option to search through the GEO database, no new datasets were found in this search, but research articles found in this search were used to inform the discussion section in this manuscript. We describe the differences and similarities between the findings of the original studies for each tissue type in *Mus musculus* and *Homo sapiens*.

### Differential gene expression comparative meta-analysis on the musculoskeletal system

**Fig. 9:**
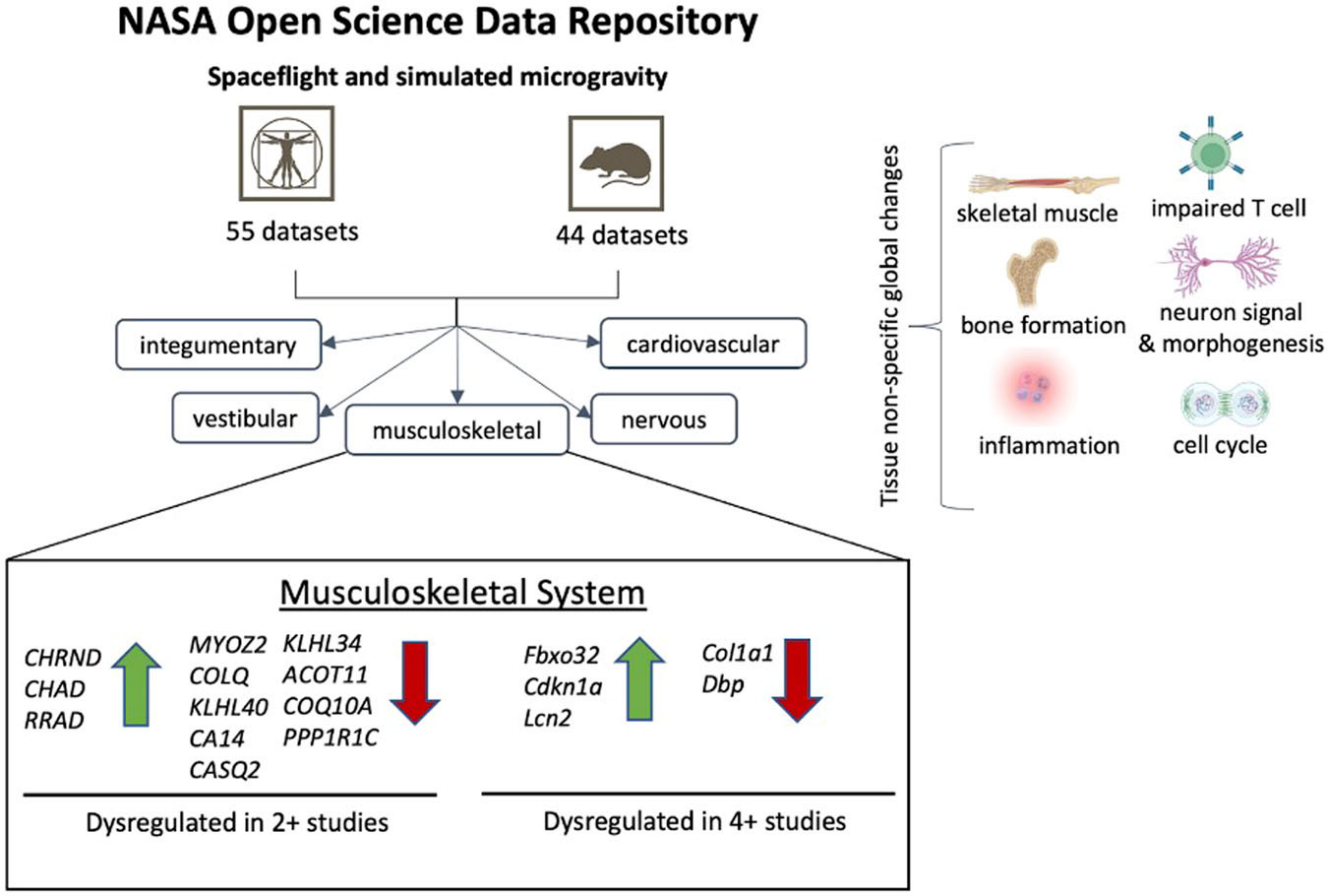
An overview of the effects of altered gravity environment on various physiological aspects in both mouse and human systems incudes musculoskeletal, immune, cardiovascular, vestibular, integumentary, and nervous system alterations. Comparative meta-analysis findings highlight certain degrees of overlap and reproducibility in genes identified as differentially expressed within musculoskeletal tissues in each species.

We also performed a comparative meta-analysis of differentially expressed genes from transcriptomics datasets between *Mus musculus* and *Homo sapiens*, specifically concentrated on the musculoskeletal system (Fig. 9). This choice was driven by the fact that the musculoskeletal system encompasses a wide range of tissue types that are highly comparable between human and mouse datasets. By focusing on this physiological system, we aimed to evaluate the consistency of findings and identify commonly upregulated and downregulated genes within each species, as well as between the two species.

To demonstrate the concordance on a gene-by-gene level between experiments we calculated Pearson correlation coefficient between gene counts in different datasets from the same tissue type. To avoid duplicate genes per experiment we calculated the average expression value for each common gene and removed null values.

To identify differentially expressed genes that are reproducible between experiments, we focused on experiments which fulfill these inclusion criteria: (1) case–control study, (2) derived from musculoskeletal tissue of the same species, (3) untreated samples, (4) microgravity vs control group comparison. We identified in each study genes which were differentially expressed between microgravity-exposed samples and control samples with an adjusted *P* value (Benjamini–Hochberg method) threshold of below 0.05 and |log2FC| threshold above 1, which reflects a substantial change in gene expression.

The data preprocessing and differential expression analysis procedures were conducted using the R programming language. Normalization was performed using the affy or oligo packages in the R Bioconductor environment^65–67^. Specifically, for the Affymetrix datasets, we employed the Robust Multiarray Analysis (RMA) algorithm through the R/Bioconductor packages affy and oligo which performs background correction, log2 transformation, quantile normalization, and the summarization of all probe sets into a single expression value for each gene. For the Agilent datasets, we performed normalization using the R/Bioconductor package limma. Following this preprocessing pipeline, each normalized dataset underwent quality control checks, incorporating an outlier removal approach. Quality control procedures were executed using the R/Bioconductor package arrayQualityMetrics, which involved generating boxplots of logarithm ratios, PCA and MA plots. Any samples identified as outliers in both MA and PCA plots during the quality control assessment were excluded from their respective datasets.

## Data Availability

Data used in the preparation during the current research article were obtained from NASA GeneLab Open Science Data Repository (OSDR; osdr.nasa.gov/bio). (Accession codes, DOIs): (OSD-33, 10.26030/7btg-6q49), (OSD-32, 10.26030/jpyz-fn46), (OSD-536, 10.26030/bg5q-t229), (OSD-232, 10.26030/9626-w275), (OSD-135, 10.26030/rjyq-x751), (OSD-396, 10.26030/ce4f-xx71), (OSD-111, 10.26030/9580-9n52), (OSD-228, 10.26030/bfmx-z866), (OSD-227, 10.26030/vk0m-0558), (OSD-21, 10.25966/c36b-3g68), (OSD-30, 10.25966/90vx-bf79), (OSD-107, 10.26030/2x2z-6w28), (OSD-324, 10.26030/s7kj-h383), (OSD-547, 10.26030/y2af-c498), (OSD-18, 10.25966/j6hy-d340), (OSD-29, 10.25966/6rr8-r017), (OSD-50, 10.26030/69kd-nx87), (OSD-61, 10.26030/2zjp-sj35), (OSD-116, 10.26030/ya5a-e896), (OSD-4, 10.25966/qq9p-pc28), (OSD-25, 10.25966/kzxa-s692), (OSD-48, 10.26030/jq04-0n51), (OSD-63, 10.26030/gsmt-8e70), (OSD-51, 10.26030/3w73-jn41), (OSD-198, 10.26030/kcrr-p336), (OSD-195, 10.26030/r6bv-rk07), (OSD-370, 10.26030/zyy0-6497), (OSD-124, 10.26030/6fwd-2p79), (OSD-546, 10.26030/mg6a-qv31), (OSD-174, 10.26030/6sg0-ng36), (OSD-118, 10.26030/rcyt-qp10), (OSD-114, 10.26030/yssj-bg68), (OSD-5, 10.25966/qq4z-4m04), (OSD-13, 10.26030/4an8-r968), (OSD-484, 10.26030/semj-9y19), (OSD-189, 10.26030/r0md-de60), (OSD-172, 10.26030/jx41-b816), (OSD-188, 10.26030/3jq1-s218), (OSD-283, 10.26030/ge2v-wr94), (OSD-297, 10.26030/bb5k-1h18), (OSD-545, 10.26030/7ta4-ga35), (OSD-54, 10.26030/8mfb-wa73), (OSD-52, 10.26030/nt3p-p547), (OSD-55, 10.26030/9thk-dv75), (OSD-56, 10.26030/nc96-xp67), (OSD-128, 10.26030/73sd-9a85), (OSD-129, 10.26030/sec3-y188), (OSD-125, 10.26030/6dnk-x507).

## Code availability

The code for the bioinformatics methods can be made available upon reasonable request to the corresponding author.
